# Deciphering the transcriptional regulation and spatiotemporal distribution of immunity response in barley to *Pyrenophora graminea* fungal invasion

**DOI:** 10.1186/s12864-016-2573-x

**Published:** 2016-03-22

**Authors:** Ahmed Ghannam, Houda Alek, Sanaa Doumani, Doureid Mansour, Mohamad I. E. Arabi

**Affiliations:** Laboratory of Plant Functional Genomics, Division of Plant Pathology, Department of Molecular Biology and Biotechnology, Atomic Energy Commission of Syria (AECS), P.O. Box 6091, Damascus, Syria; Laboratory Plant Disease, Division of Plant Pathology, Department of Molecular Biology and Biotechnology, AECS, P.O. Box 6091, Damascus, Syria

**Keywords:** *Hordeum vulgare*, *Pyrenophora graminea*, Leaf stripe, Differential display, Expressed sequence tags, Transcriptional gene networking, Callose deposition

## Abstract

**Background:**

Barley leaf stripe disease, caused by the fungus *Pyrenophora graminea* (*Pg*), is a worldwide crop disease that results in significant loss of barley yield. The purpose of the present work was to use transcriptomic profiling to highlight barley genes and metabolic pathways affected or altered in response to *Pg* infection and consequently elucidate their involvement and contribution in resistance to leaf stripe.

**Results:**

Our study examined and compared the transcriptomes of two barley genotypes using an established differential display reverse-transcription polymerase chain reaction (DDRT-PCR) strategy at 14 and 20 days post-inoculation (dpi). A total of 54 significantly modulated expressed sequence tags (ESTs) were identified. The analysis of gene expression changes during the course of infection with *Pg* suggested the involvement of 15 upregulated genes during the immunity response. By using network-based analyses, we could establish a significant correlation between genes expressed in response to *Pg* invasion. Microscopic analysis and quantitative PCR (qPCR) profiling of callose synthase and cellulose synthases revealed a direct involvement of cell wall reinforcement and callose deposition in the *Pg*-resistant phenotype.

**Conclusions:**

We have identified a number of candidate genes possibly involved in the host-pathogen interactions between barley and *Pg* fungus, 15 of which are specifically expressed in *Pg*-resistant plants. Collectively, our results suggest that the resistance to leaf stripe in barley proceeds through callose deposition and different oxidation processes.

**Electronic supplementary material:**

The online version of this article (doi:10.1186/s12864-016-2573-x) contains supplementary material, which is available to authorized users.

## Background

Barley (*Hordeum vulgare*) ranks among the most important cereals cultivated by humans in diverse environmental conditions worldwide [1]. Barley crop production is endangered by varied biotic stresses [2]. Agricultural practices directly affect crops environment. In this case crops like barley can become targets for variable biotic stresses and suffer from diseases [2, 3]. *Pyrenophora graminea* Ito & Kuribayashi [anamorph *Drechslera graminea* (Rabeneh. Ex. Schlech. Shoem)] (*Pg*) the leaf stripe disease agent is a seed-borne pathogen that systemically spreads in infected barley plants [4]. The disease is responsible for substantial reduction of barley yields in different cultivation areas [5]. The fungus survives within the kernels developing mycelia on the pericarp, but not within the embryo. When a barley seed germinates the hyphae accelerates its intercellular growth within the coleorhizae, the embryo, the roots and scutellar node, in order to establish a full-scale infection in the seedling [6]. During the early steps of colonization, the fungus behaves as a biotrophic pathogen and degrades cell-walls without causing necrosis in plant host cells [6–8]. The pathogen switches to a necrotrophic growth behavior when the fungal infection spreads into the young leaves [9]. Wide fungal growth in plant leaves causes inter-vein longitudinal necrotic stripes, as well as spike sterility which in turn causes drastic yield losses. The infected plants spread fungal spores to nearby plant spikes where a new cycle can start [5]. A limited number of studies have explored the host factors contributing to resistance to *Pg* [10–13]. Resistance to *Pg* is generally structured by a non-race specific system or major genes [6, 14]. Previous survey studies in the field have recorded a broad variability among barley responses to *Pg* infection. These ranged from highly resistant lines to highly susceptible ones [8]. This can be attributed to the genetic background of barley genotypes and *Pg* isolates [15].

An exploration of the interaction between barley and *Pg* have previously shown changes in gene expression in resistant near-isogenic line NIL3876-*Rdg2a* as a result of inoculation with the virulent isolate of *Pg*, *Dg2*. This resistance is conferred by the *Rdg2a* gene, which can arrest the fungal growth of *Dg2* isolate at the scutellar node and basal region of barley embryo provascular tissue [16]. *Rdg2a* gene, turned out to encode a protein of the CC-NB-LRR type, which confers immunity toward the *Pg*-isolate *Dg2* without the establishment of a hypersensitive response (HR) [17]. Remarkably, numerous genotypes of barley display a resistant phenotype without HR, resulting in partial or quantitative resistance [7, 12]. So far, little is known about the genetic variability/background and the mechanisms underlying the barley-fungus interactions in pathosystems other than *Dg2-Rdg2*. However, some genetic variations were found in the pathogenicity of *Pg* isolates collected from different regions of Syria, the most virulent isolate being *Pg-Sy3* [8].

Deciphering the molecular basis of more plant-pathogen interactions would significantly assist the development of new control strategies through the identification and characterization of host-plant factors and pathogenic effectors required for the infection establishment [18, 19]. Transcriptomic approaches are being widely utilized to address various biological questions and profiling the changes that take place on the genome-wide scale in response to pathogen invasion. This permits to identify genes responsive to pathogen attack or genes related to plant resistance [20]. Moreover, using transcriptomic differential screening techniques such as differential display reverse transcription-PCR (DDRT-PCR) can uncover genes with altered expression pattern, which are involved in the plant responses to pathogens. The DDRT-PCR approach, once established, is an efficient display of the whole transcript profiles in individual tissues, particularly during developmental stages or under other inducible characters [21].

In the current study, we describe a DDRT-PCR approach aiming to isolate barley genes characterized by a *Pg*-resistance specific pattern of expression while avoiding the selection of general defense-related genes. Expression of the selected expressed sequence tags (ESTs) was further investigated and related genes were characterized. Co-expression module network of resistance-related genes was generated and tested to confirm their involvement in plant immunity response against *Pg* invasion.

## Results

### Banteng genotype, but not Fourat1, confers immunity to leaf stripe independently of the Rdg2a gene

**Fig. 1:**
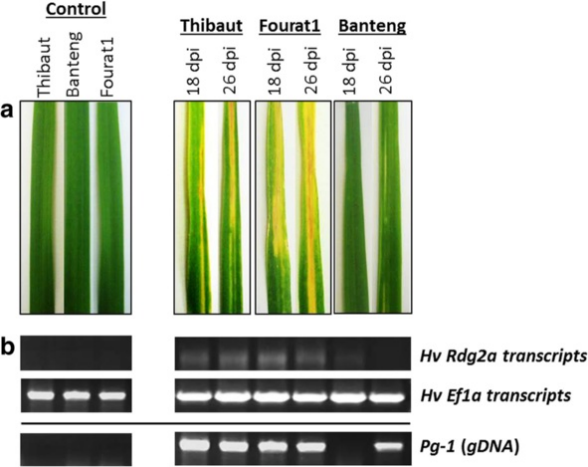
Analysis of infection on barley genotypes. **a** Seeds of Banteng, Fourat-1 and Thibaut cultivars were *P. graminea*-inoculated with isolate *Sy3* and disease symptoms were monitored at 18 and 26 dpi. **b** The upper panel represents the *Rdg2a* gene expression by RT-PCR. The middle panel represents the barley *HvEf1-α* that was used as an internal control and the *Pg-1* marker was used for estimating the fungal DNA content (*Pg*) by RT-PCR using specific primers

Two barley genotypes were used in this comparative study, a *Pg*-susceptible barley (Fourat1) with a highly susceptible phenotype (S), and a *Pg*-resistant barley (Banteng) with an immune or resistant phenotype (R). The infection evaluation was executed by inoculating the *Sy3* isolate of *P. graminea* on these two genotypes as well as on the Thibaut genotype, a positive control of resistance as it possesses the *Rdg2a* gene which is known to confer race-specific resistance to *Dg2* isolate of *P. graminea* [17]. The three genotypes were challenged for *Pg* fungal invasion for 24 days. Semi-quantitative RT-PCR was achieved using specific primers for the barley *Rdg2a* resistance gene and the *P. graminea Pg-1* gene in both pathosystems; *Pg*-S barley and *Pg*-R barley. Studied genotypes were checked on 18 and 26 days post-inoculation (dpi), which correspond to the time of the first differentiated leaf emergence and the time of symptom appearance, respectively. In the resistant genotype, plants challenged with the fungal isolate *Pg-Sy3* showed no leaf stripe symptoms at 18 dpi. This was correlated with undetectable transcripts of the fungal gene *Pg-1* signifying that no fungal mycelium was present in tested leaves (Fig. 1a, b). By the 26th dpi, symptoms started to appear with very low percentage of leaf stripe (less than 5 %) and insignificant amount of *Pg-1* transcripts was detectable when augmenting the number of PCR cycles to 29 (Fig. 1b). In contrast, noticeable necrotic symptoms and fungal transcripts were observed in the leaves of the susceptible genotype challenged with the same fungal isolate *Sy3* after 18 and 26 dpi. In the same experiment, all barley genotypes challenged with *Pg-Sy3* isolate showed no detectable levels of expression of *Rdg2a* gene, similarly to the non-inoculated control plants at 26 dpi (Fig. 1b).

Taking note of the monitored susceptibility of Thibaut genotype to the isolate *Pg-Sy3* and as the *Rdg2a* gene could confer high resistance in Thibaut genotype vis-à-vis to only *Pg-Dg2* isolate with a race-specific resistant trait [17], we can conclude that the immunity phenotype specificity of our *Pg*-resistant genotype (Banteng) to *Pg-Sy3* isolate is independent of *Rdg2a* resistance gene.

### Infection process and barley temporal dynamic response to leaf stripe fungus

**Fig. 2:**
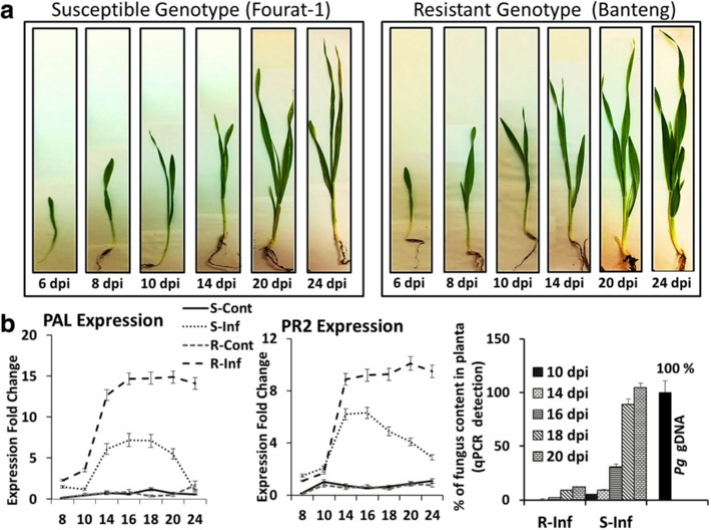
Monitoring of barley phenotypic and molecular changes. **a** temporal kinetic of infection in susceptible and resistant genotypes between 6 and 24 dpi. **b** Relative gene expression using qPCR analysis of PAL and PR2 transcripts and quantification of fungal *Pg* DNA present in R and S barley seedlings at selected time-points. Student *t* test was applied on gene expression data. Asterisks designate a statistical difference at *P* < 0.001 on each sample mean. The error bars correspond to the 95 % confidence interval calculated from the Student *t* test

Understanding the expression pattern changes and reprogramming of genes involved in perception and signaling pathways in *Pg*-resistant and *Pg*-susceptible barley during *Pg* fungal invasion requires exploring the *Pg* fungus growth rates in plant tissues and the transcriptional window of infection kinetic. In order to achieve that, we have first inspected the differential dynamics of disease development by comparing the time-window of symptoms appearance between the two genotypes (Fig. 2a). This was followed by comparing the patterns of gene expression amplitude and the temporal kinetic of plant response in both genotypes (Fig. 2b). The comparison included key genes considered as hallmarks of typical defense plant responses such as the genes that encode for Pathogenesis-Related (PR) proteins like PR2 or phytoalexin biosynthesis-related proteins like phenylalanine ammonia lyase (PAL) (Fig. 2b). A time-window between 6 dpi (early stage without symptoms) and 24 dpi has permitted the display of different plant responses to the pathogen after inoculation (Fig. 2a). Remarkably higher defense reactions were observed in inoculated *Pg*-resistant genotype compared to the inoculated *Pg*-susceptible genotype (Fig. 2b). The transcripts of *PAL* and *PR2* genes appeared to be significantly upregulated at 14 dpi and reached the optimal point of expression at 20 dpi (Fig. 2b). Upregulation of these defense-related genes in *Pg*-resistant genotype was accompanied with significant inhibition of fungal mycelial growth starting at 14 dpi, which was confirmed by the presence of genomic DNA of *Pg* fungus in plant tissues (Fig. 2b). The fungal mycelium spread within leaf tissues of *Pg*-susceptible genotype and reached 90.7 ± 1.9 and 100 ± 2.3 % after 18 and 20 dpi, respectively. In contrast, a significant slowdown of mycelium development was observed in *Pg*-resistant genotype to reach only 11.9 ± 2.2 % at 20 dpi. Our study thus indicates that 14 dpi constitutes a significant starting time-point in demonstrating the differential response of both genotypes and that the optimal time-point was 20 dpi (Fig. 2b).

### DDRT-PCR subtractive screening for immunity interactors against P. graminea

Based on the results of differential dynamics of barley genotypes response to *Pg* fungal invasion (Fig. 2a), the chief gene expression changes, which are essential for plant immunity establishment, took place in the leaf tissues between 14 and 20 dpi. At the final time-point the fungus has already colonized the plant tissues in the *pg*-susceptible genotype (Fig. 1a, b) while the defense responses had constrained the pathogen growth in the lower part of the leaf tissues in *pg*-resistant genotype (Fig. 2b). In order to identify genes that are potentially involved in the induction of barley immunity against *pg* invasion, a DDRT-PCR was performed on *Pg*-susceptible and *Pg*-resistant genotypes at 14 and 20 dpi. The subtraction of *Pg*-susceptible genes induced on the 14 or 20 dpi has facilitated the elimination of typical genes induced upon fungal infection and consequently allows the enrichment of *Pg*-resistance related genes only.

The DDRT-PCR strategy to characterize ESTs specific to *Pg*-resistance is described in Additional file 1: Figure S1. The reverse transcripts were generated from the extracted RNAs. The cDNA templates were then amplified using 29 pairs of random primers. This generated about 11000 bands called “transcript derived fragments” (TDFs). The results showed 193 TDFs with a *Pg*-defense-specific profile (PCR products present in both genotypes). While 72 TDFs showed an early (14 dpi) and late (20 dpi) *Pg*-resistance-specific profile (a PCR product presents in the infected *Pg*-resistant genotype but absent from infected *Pg*-susceptible genotype and non-infected control) (Additional file 1: Figure S1). Remarkably, the intensity of defense-related PCR products found in both genotypes was higher than that of the 72 selected TDFs. The 72 TDF bands were excised and extracted before re-amplification. The final PCR products were cloned to obtain unique sequences and eliminate duplicates. All of the 72 TDFs clones were sequenced to check for redundancy and homology. Sequence analysis of the 72 TDFs showed one to two unique sequences in each single excised band, with a mean of ~1 ± 1. This has reduced the number of retained ESTs to 54 which are induced preferentially in *Pg*-resistant genotype after inoculation with fungus. ESTs were analyzed in specific EST and GenBank databases (Additional file 2: Table S1). Selected ESTs were grouped according to their homology into five broad categories: defense/signaling/regulation (46 %), proteolysis (19 %), cell development (7 %), general metabolism (15 %) and putative/predicted/unknown (15 %) (Additional file 2: Table S1 - Additional file 3: Figure S2). Interestingly, these 54 selected ESTs were distributed among monocot plant species as follows: *Hordeum vulgare* (72 %), *Triticum asetivum* (9 %), *Oryza sativa* (9 %), *Zea mays* (6 %) and *Brachypodium distachyon* (4 %) (Additional file 4: Figure S3). Data showed several other ESTs which may have played a role in the regulation or induction of immunity response in barley against *Pg* invasion. For example, *Hv-Pg13, Hv-Pg27 and Hv-Pg30* showed significant homology with sequences encoding phosphorylated proteins like kinases, and *Hv-Pg19, Hv-Pg43 and Hv-Pg44* showed homology with sequences encoding transcription factors. Few ESTs showed homology with sequences that encode potential proteins involved in posttranscriptional regulation of signaling constituents through either the proteolysis by the ubiquitin/26S proteasome system like *Hv-Pg16, Hv-Pg18, Hv-Pg37* and *Hv-Pg49* or through defense-specific enzymatic activity (*Hv-Pg21, Hv-Pg7, Hv-Pg10* and *Hv-Pg26*). A considerable part of the ESTs population, almost 15 %, showed no homology with databases or homology with unknown proteins. The induction of homologues in other biotic treatment systems was the first indication on the importance of such candidate genes (Additional file 2: Table S1).

### Temporal patterns of differentially expressed genes (DEGs) in immunity response against P. graminea

**Fig. 3:**
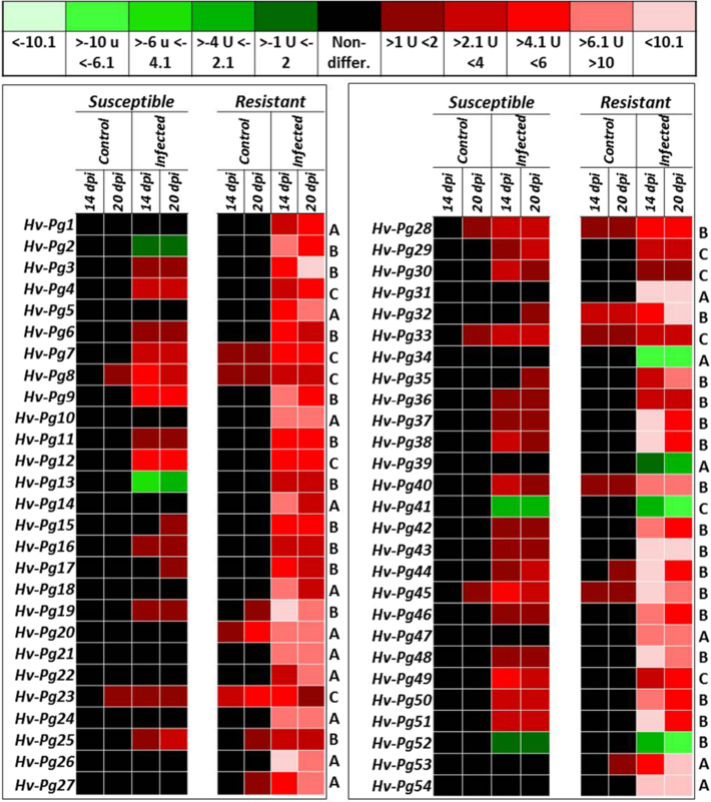
Temporal expression of ESTs (genes) selected by RT-PCR differential screening. After extraction of total RNA from plant tissues at 14 and 20 dpi, reverse-transcripts were produced for carrying-out qPCR analysis. Control to normalize amplification was run with the *Ef1-α* specific primers. Transcripts were analyzed using comparative Ct method. Data are presented in color scales: five shades of red from dark red to light red for upregulated genes and five shades of green for the downregulated genes. Non-differential expression is mentioned in black. The value of relative quantification (RQ) from qPCR was represented by the ladder of both colors (top panel)

Gene expression analysis were performed *in planta* using qPCR amplification at the same time-points used in DDRT-PCR approach to confirm the temporal specific expression pattern of selected *Pg*-resistance-specific genes in the two barley genotypes upon inoculation with *Pg* fungus. Eleven characteristic categories of expression fold change (eFC) were identified when investigating the expression pattern of 54 selected genes (Fig. 3). The expression levels were coded using different color shades (Fig. 3). Upregulation of expression was marked in five shades of green, while the downregulation of expression was marked in red. Color intensity correlates with the level of expression. When no difference in expression was detected, black color was used (ladder in Fig. 3). Analyzing the data on both the 14 and 20 dpi showed that both transcriptome of susceptible and resistant barley genotypes were exposed to drastic changes in response to *Pg* invasion (Fig. 3). Results highlighted 54 characteristic genes that were sorted into three representative groups. Group A contains genes that show a specific change in expression pattern (expressed or repressed) only in *Pg*-resistant genotype. Group B contains genes that are preferentially expressed, that are genes showing an expression change in resistant genotype, weak and delayed expression in the susceptible genotype but not in controls of each genotype. A general defense-related gene expression profile forms group C, where genes were expressed similarly in the tissues of both genotypes but not in controls. The largest number of genes (27 genes) is contained in group B, while group A and group C have fewer numbers, 17 and 10 respectively (Fig. 3). Remarkably, the expression level of the majority of group B genes was very low in the susceptible genotype compared to the resistant genotype.

Moreover, some of the genes in group B and C (*Hv-Pg8*, *Hv-Pg23*, *Hv-Pg28, Hv-Pg33* and *Hv-Pg45*), showed a faint constitutive background expression in the controls of the susceptible genotype. However, the genes (*Hv-Pg7, Hv-Pg19, Hv-Pg20, Hv-Pg25, Hv-Pg27, Hv-Pg32, Hv-Pg44* and *Hv-Pg53*) with a faint constitutive background expression in controls of resistant plants were more important and were considered in the semantic analysis of the plant immunity responses (Fig. 3). Notably, only 14 genes (*Hv-Pg3*, *Hv-Pg19*, *Hv-Pg26*, *Hv-Pg31*, *Hv-Pg32*, *Hv-Pg37*, *Hv-Pg38*, *Hv-Pg43*, *Hv-Pg44*, *Hv-Pg45*, *Hv-Pg48*, *Hv-Pg51, Hv-Pg53* and *Hv-Pg54*) have shown high upregulation (more than 10 folds) in the resistant genotype with no or very low expression in susceptible genotype. Predictably, the majority of these genes are present in the early time kinetic. Hence, genes with a specific pattern of expression (A and B groups) represent ~80 % of the studied genes which confirms the efficacy of this screen. Moreover, significant correlation is observed between expression levels of genes from “A and B” groups and the intensity of the 72 TDFs. As for downregulated genes, two genes were present in the resistant genotype (*Hv-Pg34* and *Hv-Pg39*) and two (*Hv-Pg41* and *Hv-Pg52*) were present in both genotypes.

### Spatial patterns of DEGs in immunity response against *P. graminea*

**Fig. 4:**
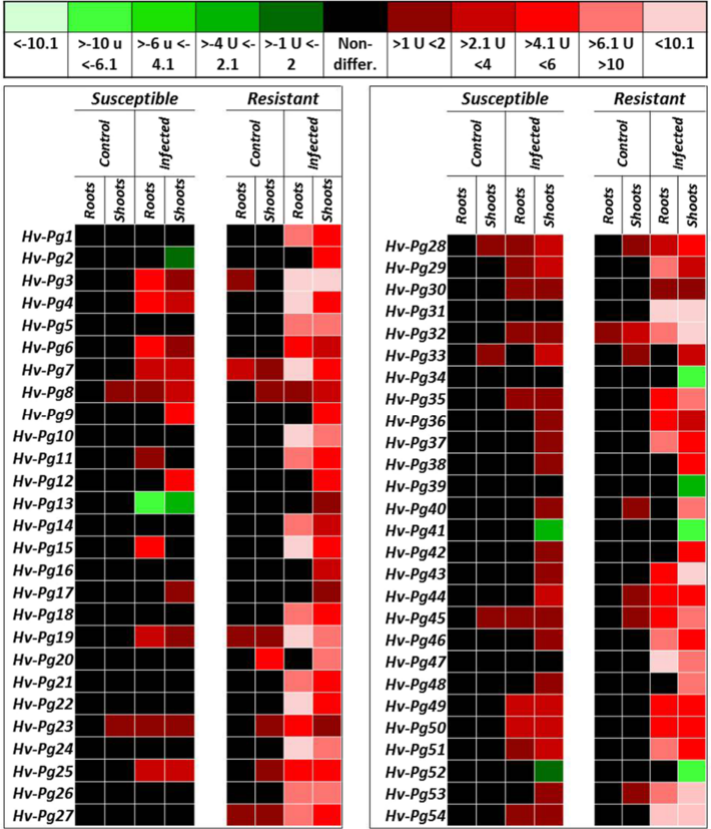
Spatial expression of ESTs (genes) selected by RT-PCR differential screening Barley seedlings were inoculated for 14 and 20 dpi. Roots or shoots of all inoculated plants mixed together in pools representing14 and 20 dpi together. qPCR runs were performed and illustrated like presented in Fig. 3

Based on previous reports and information on *Pg* fungus and its growth in all parts of infected plants, we analyzed the spatial distribution of expression of the same 54 selected genes. This was performed on shoots and roots of tested plants using a pool of equal RNA amounts from 14 and 20 dpi samples (Fig. 4). The goal of this assessment was to better understand the pattern of the 54 inspected genes in the whole plants against *Pg* fungal invasion. As expected, all analyzed genes showed differential expression in shoots and roots, while the majority had conserved expression levels in the resistant genotype.

Remarkably, the number of genes with high upregulation levels (more than 10 folds) in roots of the resistant genotype was contrasted with those of highly upregulated genes in temporal kinetic and restricted to 10 genes (*Hv-Pg3*, *Hv-Pg4*, *Hv-Pg10*, *Hv-Pg15*, *Hv-Pg19*, *Hv-Pg22*, *Hv-Pg24*, *Hv-Pg31*, *Hv-Pg47* and *Hv-Pg54*). Interestingly, most of the highly upregulated genes in the temporal kinetics in resistant genotype at 20 dpi (Fig. 3) were visibly upregulated in roots within an eFC between 6 and 10 (Fig. 4).

### Global effect of Pg invasion on differential gene expression of barley genotypes

**Fig. 5:**
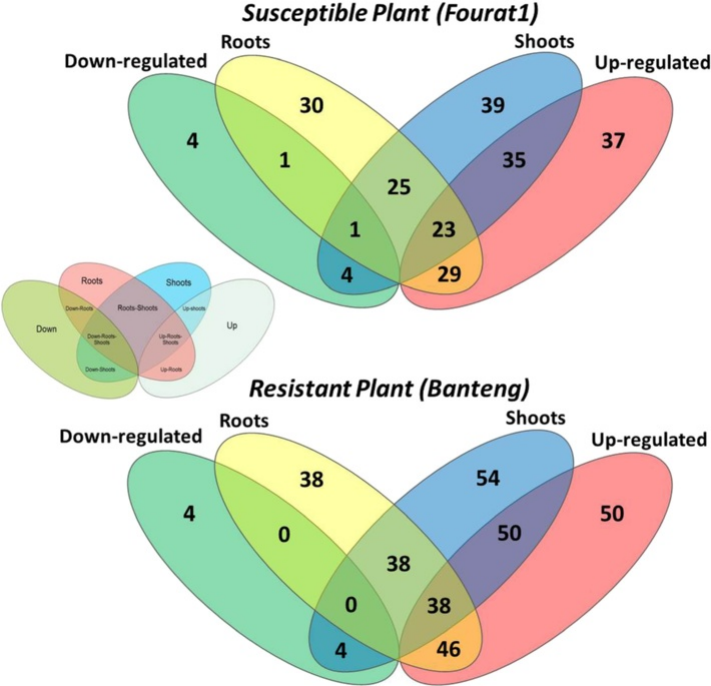
Numbers of differentially expressed genes (DEGs) in different barley genotypes tested in roots and shoots. The Venn diagram shows the number of genes up- or downregulated in root and shoot tissues in response to *Pg* inoculation at levels of 2 folds or more and a *P* value < 0.05

Upon *Pg* inoculation, the resistant genotype showed the induction of 54 genes in shoots highlighted by the qPCR results and which were possibly involved in plant immunity responses to the infection of leaf stripe fungus. However, in the spatio-temporal analysis, all selected genes were analyzed in at least one of the two tested genotypes per time point and in one spatial distribution combinations “roots/shoots”. Remarkably, 50 of them were significantly upregulated by the *Pg* infection. However, of 46 upregulated genes from the resistant genotype background, 38 genes were induced in both roots and shoots and only 4 genes were downregulated and the expression was spatially distributed only in shoots (Fig. 5).

Per contra, the susceptible genotype showed an induction of 39 genes in shoots, of which 35 genes were upregulated upon *Pg* infection. An induction of 30 genes was noted in roots of the susceptible genotype and 29 of these were upregulated. Data also showed the induction of 23 genes in both roots and shoots of susceptible genotype. However, only one gene of the downregulated genes in the susceptible genotype was downregulated in roots and shoots tissues (Fig. 5). Furthermore, the number of common genes expressed in roots and shoots was divergent between resistant and susceptible genotypes. Consequently, data showed a partial spatial expression match between both tissues (Additional file 5: Figure S4).

### Gene ontology of derived cDNA sequences

**Fig. 6:**
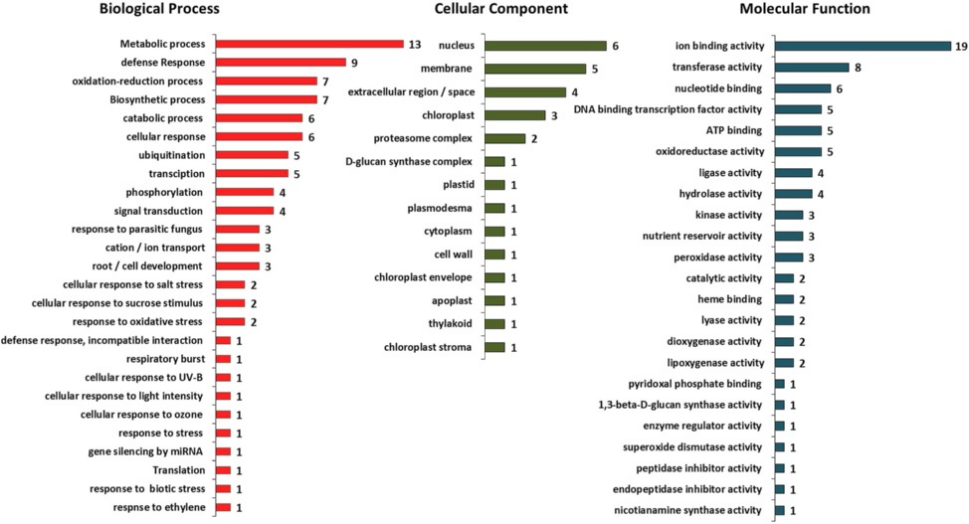
Classification of Gene Ontology (GO) analysis of selected DEGs after inoculation with *Pg*. A total of 54 genes were categorized in three groups after GO enrichment: Molecular function, Cell component and Biological process

Gene Ontology (GO) analysis of the matched ESTs has identified several gene annotations that were categorized into different GO groups (Fig. 6). Some of gene categories are partially redundant, which led to categorize them into more than one group. In the molecular function category, genes assigned to the ion binding, transferase activity or DNA binding groups were highly enriched. In the cellular component category, genes in the “nucleus”, “membrane” and “extracellular region” groups were the most abundant. In the biological process category, the “metabolic process”, defense response”, “oxidation process” and “biosynthetic process” groups contained the largest number of genes (Fig. 6).

### Semantic analysis and co-regulation of immunity response in DEGs

**Fig. 7:**
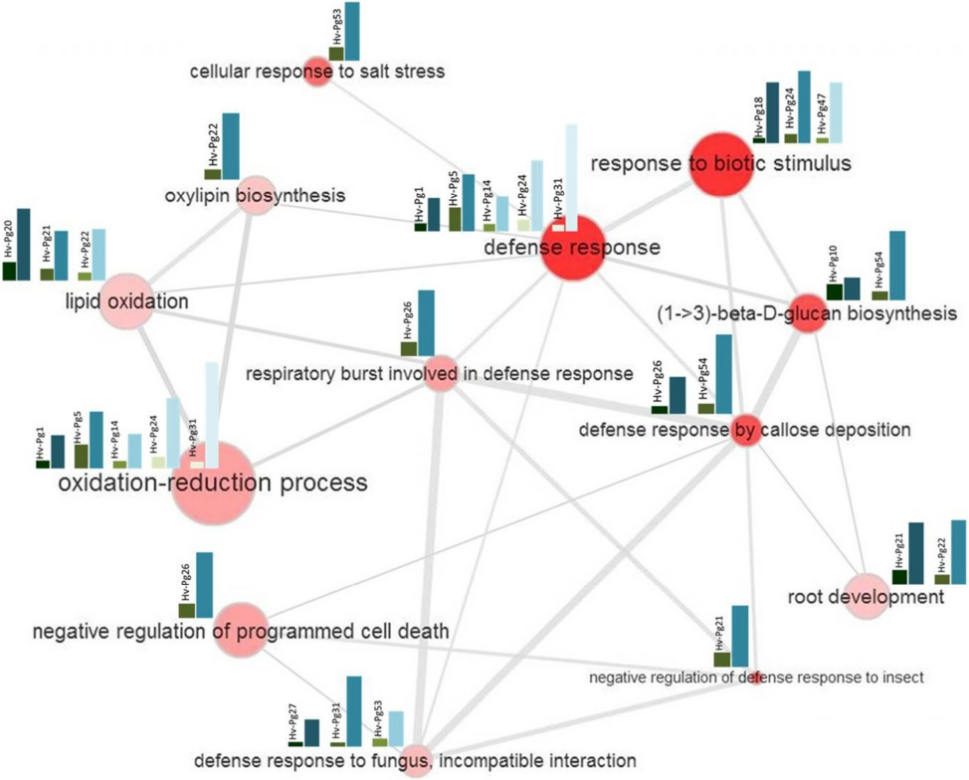
Functional network of enriched gene set in the resistant genotype. The 15 genes selected by qPCR analysis for their resistance-specific pattern of expression, were annotated using Blast2GO and the functional GO terms were manually selected. Network map of GO terms interactions was generated using Cytoscape. The network-interacting genes annotated with a particular term are represented by nodes. Nodes are sized according to annotated/enriched gene number. The connections between nodes means at least one gene is shared between enriched genes. The thinness of links represents the significance of linking between nodes. Gene expression analysis using qPCR of each annotated gene was incorporated in the network to represent the behavior of each of them in both susceptible and resistant genotypes

In order to understand the impact of fungal invasion on barley plants immunity and gene expression patterns during establishment of infection, the set of upregulated genes in group A was investigated for a potential networking or functional connections between the related assigned-annotation terms. This set contains 15 genes, which are mainly DEGs with no expression in susceptible genotypes (*Hv-Pg1*, *Hv-Pg5, Hv-Pg10, Hv-Pg14, Hv-Pg18, Hv-Pg20, Hv-Pg21, Hv-Pg22, Hv-Pg24, Hv-Pg26, Hv-Pg27, Hv-Pg31, Hv-Pg47, Hv-Pg53* and *Hv-Pg54*). The plant cDNA sequences of these 15 genes, which were derived from our DDRT-PCR approach, were deposited at GOslim for analysis to extract implicit semantic relationships between genes and functions based on the data set of databases/semantic relationships between GO terms. A starting model of functional annotation network map was built for analyzed genes including values of qPCR analysis in roots and shoots. This network represented the upregulated genes of Group A (Fig. 7); where the different functional annotation terms were represented by nodes and the size of each node was calculated according to the number of genes associated with this annotation. A line linking two annotation terms indicates that at least one gene is assigned to both functional annotations.

As illustrated in Fig. 7, the presented gene set contains genes with variable functions, going from biotic stress response to root development. The produced network mapping allowed us to highlight semantic connections and functional grouping of genes that are differentially expressed (DEGs) in our experimental state of immunity response. Connecting these genes together in a functional network; by considering all data of enriched GO terms and by including values of our qPCR gene expression study; should be informative to understand barley immunity co-regulation against *Pg* infection. Oddly, the strongest relationships were established among the following: defense response, defense response by callose deposition, 1- > 3 beta-D-glucan biosynthesis and response to biotic stimulus where the size and color of nodes and the width of connections represents the strength of the link between these annotated functional terms. In the co-regulation network, relation of callose deposition mechanism with the respiratory burst and different oxidation processes was demonstrated similarly to the immunity response of barley plants against *Pg* attack. This relation is illustrated through significant links and notable nodes representing the potential participation of oxidation process in the establishment of the plant responses. Moreover, the representation of the qPCR expression profile of root and shoot candidate genes within the co-regulation network module, was expressed the number of genes involved in each annotated molecular function. In our network, we observe the prominent representation (as deduced from gene number and eFC) of genes potentially involved in immunity process in the resistant genotype (*Hv-Pg*5, *Hv-Pg*18, *Hv-Pg*26, *Hv-Pg*31, *Hv-Pg*47, *Hv-Pg*53 and *Hv-Pg*54) (Fig. 7).

### Cell wall fortification pathways are highly responsive to Pg attack

**Fig. 8:**
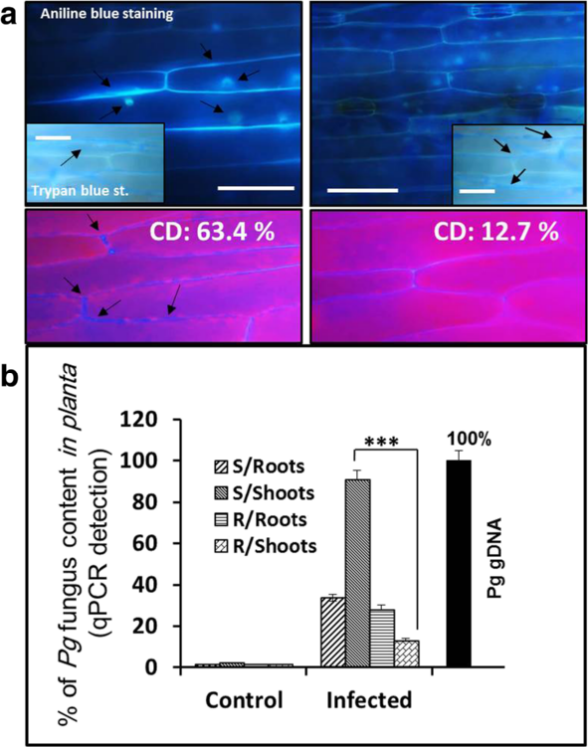
Elevated callose deposition in resistant genotype prevents *Pg* fungal growth but not susceptible genotype. Tests were performed at 14 dpi. **a** Leaves were stained with aniline blue to visualize callose deposition by blue fluorescence using florescence microscopy (upper panel). Relative fluorescence intensity emitted by aniline blue-stained callose depositions (CD) was calculated (lower panel) on photographs taken under UV filter. The average of 10 tissue samples of each category is presented as a percentage. Scale bar = 20 μm. Samples with mycelium and second hyphae (or control tissues) were also washed off, stained by trypan blue and passed on optical microscope to visualize the presence of fungus. **b** Quantification of fungal *Pg* DNA presence in roots and shoots of resistant and susceptible plants inoculated in liquid culture for 10 days was performed using qPCR. Student’s *t* test was applied and asterisks indicate a statistical difference at *P* < 0.001 on each sample mean. The error bars correspond to the 95 % confidence interval calculated from the test

To inspect the real contribution of genes selected from the co-regulation network, which present a crucial role of callose deposition in the immunity response of barley to *Pg* attack, aniline blue staining was used. This specific staining allows the visualization of callose through fluorescence microscopy. In resistant plants cultivated in liquid medium, the incline of the primary leaf tissue showed first callose deposition at 10 dpi. Then, the optimal callose deposition was detected at 14 dpi when a callose deposition core walled by a ring of callose plackets was apparent in the resistant plants. The shape and diameter of these depositions indicated a strong reaction of the underlying mesophyll plant cells against the fungal attack. Thereby, the accumulation of callose plackets formed connected callose patches in these challenged plants. However, very small callose depositions were observed in susceptible plants at 14 dpi. The quantification of callose depositions (CD) was performed by taking digital photographs under the fluorescence microscope UV filter and counting the number of blue pixels (callose intensity). Considering the same kinetics in resistant and susceptible plants, the CD counts in the susceptible plants at 14 dpi were limited to 12.7 %, whereas, in resistant plants, the CDs phenomenally increased up to 63.4 % (Fig. 8a).

Trypan blue-stained leaves were also microscopically analyzed on the same time frame (14 dpi) to investigate the density of hyphal growth. At 14 dpi, *Pg* fungus started to form a dense hyphal network in susceptible plants. Per contra, the quasi-absence of hyphal network in resistant leaf tissues was dominant in the whole surface of the first leaf (flag) (Fig. 8a). This may indicate that an active resistance reaction was initiated during infection with *Pg*. This was accompanied with a significant inhibition of fungal mycelium progressive growth starting at 14 dpi, which was confirmed on shoots and roots of resistant plants by qPCR to reach a relatively low growth percentage of only 9.7 ± 2.1 and 22.4 ± 0.7 %, respectively. In contrast, the fungal hypha largely spread in leaf tissues of susceptible genotype and reached 91.3 ± 1.3 and 38.2 ± 2.2 % in shoots and roots, respectively (Fig. 8b).

### Expression and phylogenetic analysis of callose deposition-related genes

**Fig. 9:**
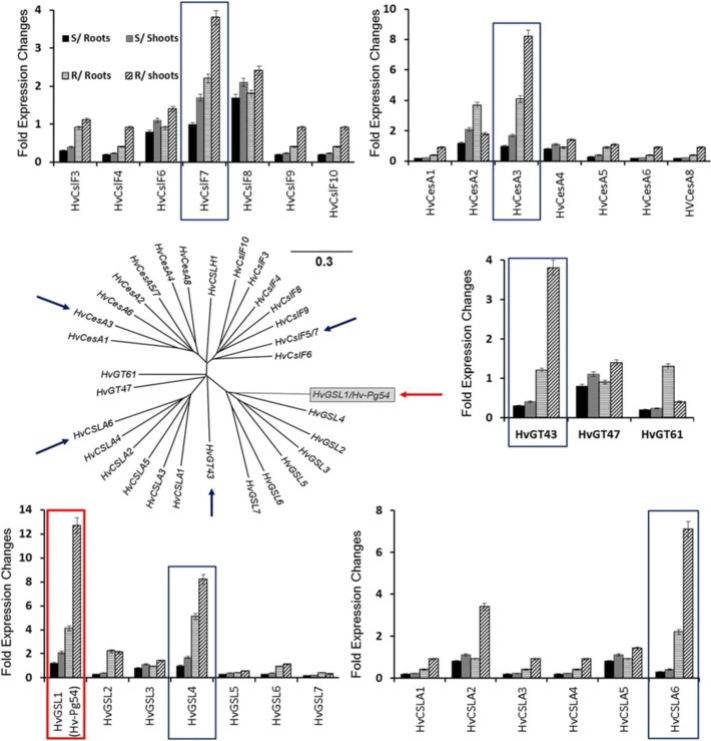
Expression levels of callose deposition related gene families after inoculation with *Pg* fungus. Phylogenetic relatedness of the barley callose synthase, cellulose synthase and related gene families to callose formation: *HvGSLs, HvCSLAs, HvGTs, HvCesAs* and *HvCsIFs* including *Hv-Pg54*. Phylogenetic tree was generated with the clustalX program and is based on nucleic acid sequences. The relative gene expression of transcripts of all genes from the five gene families was analyzed using comparative Ct method in roots and shoots of resistant and susceptible inoculated plants at 10 dpi. The error bars correspond to the calculated 95 % confidence interval

The expression levels of all barley genes potentially involved in callose deposition, were evaluated in both shoot and root tissues of both barley genotypes, by qPCR. Sequences of 30 database available genes were grouped in five gene families (*HvGSLs, HvCSLAs, HvGTs, HvCesAs and HvCsIFs*). The data regarding the *HvGSLs* gene family indicated that the expression of *HvGSL1* (also selected in our DDRT-PCR screen under the name *HvPg54*) and *HvGSL4* was remarkably increased in shoots of resistant plants after *Pg* infection to reach 12.9 and 8.2 folds respectively. Whereas, the upregulation expression levels of *HvGSL2, HvGSL3, HvGSL5, HvGSL6* and *HvGSL7* were not significant after 14 dpi (ranged between 0.1 and 2 fold). Moreover, *HvGSL1* and *HvGSL4* showed significant induction in roots too (4.1 and 5.3 folds respectively). Concerning the second family *HvCSLAs*, only *HvCSLA6* was clearly upregulated in shoots of resistant plants (7.8 folds) compared to control, with a small significant induction in roots (2.2 folds). Only one member of the third gene family, the *HvGTs*, showed a significant upregulation (3.8 folds) in shoots of resistant plants and that gene is *HvGT43*. As for the other gene families, only *HvCsIF7* and *HvCesA3* were significantly upregulated in shoots of the resistant plants (3.9 and 9.1 folds respectively) and only *HvCesA2* was upregulated (4.0 folds) in roots without significant expression in shoots (Fig. 9). The phylogenetic analysis of all genes that were subjected to the gene expression study has demonstrated the apparent grouping and homology between members of same family but divergence between gene families. This may indicate the synergetic participation of genes (*HvGSL1*, *HvGSL4, HvCSLA6, HvGT43, HvCsIF7* and *HvCesA3*) involved in the deposition of callose after *Pg* infection (Fig. 9). *HvGSL1* was the most importantly upregulated gene in both parts of the resistant plants suggesting its crucial involvement in the induced phenomena.

## Discussion

Plants developed different defense strategies to fight against pathogens. This involves complex mechanisms of interaction, which are divided in two types: PAMP-Triggered Immunity (PTI) and Effector-Triggered Immunity (ETI). PTI is the earliest response of plant under attack, when host receptors recognize the pathogen-derived PAMPs (Pathogen-Associated Molecular Pattern), whereas ETI is prompted by the interaction between a pathogenic effector and a ‘Resistance’ protein [22, 23]. The two immune systems result in a non-host resistance (considered as PTI), and partial or qualitative resistance (considered as ETI). In their most recent publications, Niks and Marcel [24, 25] may explain the partial resistance phenomenon by the variation in the capacity of pathogen effectors to suppress PTI. In cereal crops, the barley-*Pg* (barley leaf stripe) is an intriguing pathosystem of PTI reaction.

Transcriptomic is a potent method for the large-scale analysis of such interactions between plants and pathogens. The DDRT-PCR strategy was employed in our study to characterize ESTs with a *Pg*-resistance-specific expression pattern. These potentially selected ESTs seem to be implicated in the barley immunity response against *Pg* fungal invasion when the *Dg2*/*Rdg2* system is absent. In this study, we have presented a complex screen aimed to specifically identify such genes by excluding general defense-related genes induced in all genotypes, including susceptible genotype, like PAL and PR2 proteins [21]. Using 29 primer combinations, ~ 8000 DDRT-PCR bands were profiled and analyzed from denaturing gels. ESTs were found to represent up to ~10,000 unique mRNAs, if we consider that a DDRT-PCR band contains ~1 ± 1 unique sequence(s). Seventy-two DDRT-PCR bands showed a *Pg*-resistance-specific or *Pg*-resistance-preferential profile and all were of low abundance indicating an efficient screen strategy. Out of the 72 bands, 54 unique ESTs were selected after sequencing. Sequence analysis revealed that some ESTs could be considered as general defense-related genes.

The quantitative real-time PCR approach followed in our study to investigate the expression profiling of candidate genes has permitted us to study the dynamic changes in gene expression over a time course. The stimulation and signaling pathways of immunity-related genes that are specific to the interaction between Barley and *Pg* fungal pathogen require up- and downregulation of numerous genes. We are primarily interested in all genes obtained from the DDRT-PCR approach with a focus on genes whose expression might be used to identify the immunity response of the resistant genotype. By investigating the temporal distribution of expression profiles of the 54 genes *in planta*, we have confirmed the *Pg*-resistance-specific or -preferential expression pattern of 38 ESTs representing ~ 0.47 % of all screened transcripts. These 38 ESTs with a confirmed resistance expression pattern were retained in two groups. Group A contains 17 genes out of 38 (39 %), which exhibited a resistance-specific pattern with no expression detected in infected susceptible plants or controls. This high number of specific selected ESTs shows the efficacy of our subtractive screen. Genes that are up or downregulated by infection in resistant plants only are of particular interest because one or more of them may participate in inducing the immunity response mediated by leaf stripe resistance. Moreover, the integration of a susceptible genotype in our screen helped augmenting the significant number of ESTs with a *Pg*-resistance-specific pattern of expression. The cDNA-AFLP and subtractive hybridization (SSH) analysis of barley NILs (Near Isogenic Lines) performed by Haegi et al. resulted in the identification of only five SSH and 171 cDNA-AFLP host genes whose expression in barley embryo is up or downregulated by leaf stripe inoculation [16]. Of all isolated differential transcripts, *Rdg2a* encoding a CC-NB-LRR protein was the most important gene involved in immunity to leaf stripe fungus [16]. Regardless of its resistance conventional structure, *Rdg2a* can mediate immunity of barley plants in the absence of hypersensitive response [17].

The spatial expression pattern of our DDRT-PCR-selected 54 genes between roots and shoots has also confirmed the specificity of genes from Group A, even though there was a differential manifestation of spatial distribution in the expression of *Pg*-responsive genes between both genotypes and in the each genotype between roots and shoots.

Pathogen-induced genes that have been identified exclusively during this study in resistant plants have been annotated mainly for defense responses, oxidation-related, callose deposition GO terms. These functional annotations indicate their potential involvement in the PTI immunity response of resistant plants [26]. Our results emphasizes that, when *Rdg2a resistance* gene is genetically absent*,* barley plants still harbor immunity responses similar to *R*-gene-mediated responses as demonstrated by a significant up-regulation of genes involved in oxidative burst and callose deposition processes.

In order to decipher the functional signaling networks controlling resistance to *Pg* infection, we have produced a robust co-expression data module in the resistant genotype at 14 dpi, which is the most critical time-point during *Pg* invasion (Fig. 2). The module includes the 15 upregulated genes from Group A. However, we understand that host transcriptome adaptation/re-modeling in response to pathogen invasion is a dynamic process and that the immunity-related genes react to pathogen effectors’ presence on different time windows and in variable response amplitude. Therefore, it should be recognized that the defense reaction synopses built on the transcriptional data of only the 15 *Pg*-resistance-specific genes identified here only represent a snapshot of a complex biological process. Understanding the complex regulatory machineries taking place during these reactions against *Pg* should include a more inclusive analysis that involves sampling at multiple time-points covering the whole *Pg* infection course. Our results show that the strongest networking relationship in the generated module was callose deposition-related genes and oxidation-related genes. This may suggest that these functions are strongly associated with the immunity response of the resistant genotype to *Pg* infection through their contribution in the cell wall re-modeling.

Cell walls are the first barricades against pathogens, they respond to localized stress by directly deposing cellular components onto the inner surface. It has been suggested that the cell wall alteration provoked by pathogenic fungi might represent a general disease resistance mechanism through interference with the invading pathogens [27, 28]. Previously published microscopic investigations on resistance responses according to pathogen developmental phases have signposted that piercing wall of plant cell by *Pg* fungus is the most critical point determining the outcome of infection [16]. Reducing the haustorium formation by pathogenic fungi is tightly associated to pre-haustorial resistance in host plant cells. Unsuccessful attempts are typically related with cell wall reinforcement by callose deposition [29]. Our microscopic observations indicate that cell wall modifications take place during the immunity response of resistant genotype only. Some gene inductions by *Pg* were consistent with triggering cell wall alteration mechanisms. Induction of callose synthase and peroxidase genes was observed in resistant genotype only. Callose is a major constituent of papillae formations on the inner side of cell walls in response to challenge by the pathogen [30] and oxidation-related proteins like peroxidases are involved in lignification and cross-linking of phenolic compounds, proteins and carbohydrates [31]. The cell-wall reinforcement is similarly associated with the induction of reactive oxygen species (ROS). In the present study, genes encoding hydrogen peroxide-producing enzymes like oxidase were found to be induced in resistant plants. Quantitative analysis of transcripts of known gene families involved in callose deposition in barley, revealed the contribution of five of them in the immunity response to *Pg* attack. This confirmed the hypothesis of direct involvement of cell wall modification in resistant plants.

Characterizing genes specifically expressed during resistance responses remains a necessity for understanding the molecular mechanisms of the immunity response induction/regulation. So far, it is not clear if the inspected genes in the current study are the only genes linked to the induction/regulation of the immunity response in the resistant genotype. Further functional studies of gene products will be necessary via functional genomic and reverse genetics approaches in sense and antisense transgenic plants and by using virus-induced gene silencing or RNAi technologies. They should provide crucial evidences about the role of such genes in the regulatory mechanisms leading to plant resistance.

Moreover, to better understand the molecular basis of manifested partial resistance of Banteng against *Pg* fungal invasion, genomic locations that contain partial resistance loci should be characterized by QTL and expression QTL (eQTL) analysis. This could distinguish between the presence of a *cis*-acting regulatory polymorphism in the gene (*cis*-eQTL) and the location of *trans*-acting regulators (*trans*-eQTL). These elements may control the expression of a number of genes elsewhere in the genome. Furthermore, allele-specific expression (ASE) should be analyzed to assess the frequency of *cis*-acting regulatory variation and the effects of genetic background, developmental differences and *Pg* invasion/infection on allelic expression levels. These envisioned eQTL and ASE analysis will also provide the possibility of correlating observed variation in the abundance of mRNA transcripts with variations observed in simple or complex phenotypes. This is potentially an efficient route towards unraveling the molecular basis of phenotypic diversity.

## Conclusions

In this study, we applied comparative transcriptomic profiling to advance the understanding of the molecular basis of barley response to *Pg* fungus infection. The generation of RNA-seq data from infected host-plants of both genotypes has revealed a number of new DEGs that are possibly involved in the interactions between barley and *Pg* fungus. The comparative spatiotemporal analysis of selected DEGs has enabled us to identify genes that changed over the course of infection in the real-time context of a spatiotemporal dynamics of biological system, and to a much greater depth and sensitivity than it has been previously reported. This study permitted us to reveal 15 DEGs specifically upregulated in *Pg*-immunity response. In addition to providing robust sets of DEGs markers for distinguishing resistant genotypes from susceptible, the network-based analyses allowed to establish a highly specific correlation between genes expressed in response to *Pg* invasion. Microscopic and molecular analysis has confirmed that the resistance to leaf stripe in barley is coordinated and mostly executed by callose deposition and oxidation process involved in cell wall reinforcement. Furthermore, our study provides a global and more clear picture of the transcriptomic signature providing additional insights into how *Pg* pathogens are able to evade host defenses and modulate biological functions of the host-plant cell in order to establish the infection and cause disease. Moreover, this illustrative picture of plant transcriptome changes generates new understanding of how certain barley genotypes can evade infection by reinforcing their host defense systems.

## Methods

### Plant material, Pathogen isolates and inoculations

Studies were carried out by comparison of two varieties/genotypes of barley Banteng and Fourat-1. The variety Banteng was previously demonstrated to have some resistance qualities to all *Pg* isolates originated and isolated from Syrian barley fields [7, 8]. Fourat-1 is a universal susceptible variety for all Syrian isolates. In addition, the variety Thibaut was used as a positive control, since it is resistant only to *Dg2* isolate of *P. graminea* through the *Rdg2a* gene, which is known to express race-specific resistance.

In a previously described monoconidial isolates collection, the *Pg-Sy3* isolate was the most virulent of isolates collected between 2000 and 2002 from naturally infected barley in different regions of Syria [7, 8]. The fungal mycelia were transferred from a stock culture into Petri dishes containing potato dextrose agar (PDA, DIFCO, Detroit, MI, USA) supplemented with 13 mg/l kanamycin sulfate and incubated for 10 days at 20° ± 1 °C in the dark. Barley seeds were inoculated using the method described by Haegi et al. with slight modifications [16]. Briefly, fifty seeds of each barley variety were surface-sterilized with 70 % ethanol for 30 s and 5 % sodium hypochlorite (NaOCl) for 5 min. Seeds were then well rinsed three times with deionized water and incubated in Petri dishes containing an actively growing mycelium cultured on PDA medium. After 14 days of incubation in the dark at 6 ± 1 °C, the emerged seedlings were transplanted into 12-cm diameter pots and grown in a growth chamber under controlled conditions. All plants were maintained at a temperature of 16 ± 1 °C with a photoperiod of 10 h (for daylight) and 12° ± 1 °C (for night) and 70–80 % relative humidity. Uninfected control seeds were germinated under similar conditions but without fungus. Plant tissues were collected according to the kinetic of each experiment and kept at −80 °C for RNA extraction.

### RNA extraction and cDNA synthesis

The extraction of total RNA was performed from fresh tissues in a Trizol® extraction buffer (InVitrogen®, Carlsbad, USA) according to the manufacturer’s instructions. Reverse transcription was carried out on 2 μg of isolated total RNA for synthesizing cDNA populations from all treated plants by using Superscript III (InVitrogen®, Carlsbad, USA) as described in the manufacturer’s protocol.

### DDRT-PCR and TDF isolation

For the differential display screening, we used the DDRT-PCR Clontech kit (Palo Alto, CA, USA) was used as described in the manufacturer’s protocol concerning the reverse transcription, the RT-PCR and the polyacrylamide gel analysis. The several selected primer couples were utilized from the kit according to the following combinations: P1/T1, P1/T2, P1/T4, P2/T1, P2/T2, P2/T3, P2/T4, P3/T1, P3/T2, P3/T3, P3/T4, P3/T5, P3/T6, P4/T3, P4/T4, P4/T5, P5/T5, P5/T6, P6/T3, P6/T5, P6/T6, P7/T5, P7/T7, P7/T9, P8/T5, P8/T7, P9/T5, P9/T7, P9/T9. After Sliver staining of the acrylamide gels, bands showing differential pattern between compared samples were excised. Isolated bands were re-amplified and PCR products were purified as described by Ghannam et al. [21]. TDFs (transcript derived fragments) remaining after re-amplification step were inserted into pGEM-T Easy plasmid (Promega, Madison, WI) or Topo II (InVitrogen®, Carlsbad, USA).

### Sequence analysis

Sequencing was performed using the BigDye sequencing kit (applied Biosystems, Foster City, CA) and the sequencer ABI PRISM 3130 (Applied Biosystems), with universal or DDRT-PCR primers. Sequence similarity analyses were performed by matching our sequences against GenBank (nr) and EST (dbEST) databases using BLASTn and BLASTx algorithms [32] at the NCBI websites (National Center for Biotechnology Information).

### Gene expression analysis by semi-quantitative RT-PCR

PCR was performed according to this thermocycles: 94 °C for 1 min, 55–58 °C for 1 min (adjusted annealing temperature for each gene) and 72 °C for 1 min, respectively, for 25–33 cycles. EF-1α gene was used as a control reference gene to normalize RT-PCR amplification (Additional file 6: Table S2). 25 cycles-PCR reactions for the reference gene were performed on 5 dilutions of cDNA which lead to linear amplifications related to RNA quantities. Same reactions were also performed to assess *HvRdg2a* and *Pg-1* using gene-specific primers (Additional file 6: Table S2). The Bio-Rad Quantity One software and the Bio-Rad GelDoc was used to quantify PCR products on 1 % ethidium-bromided agarose gel.

### Quantitative Real-time PCR expression analysis (qPCR)

Synthesized cDNAs from RNA samples of seedling leaf and root tissues were used for qPCR analysis to validate the expression patterns detected by the differential display. Quantitative Real-Time PCR was performed using StepOne® PCR Real-Time machine (Applied Biosystems, Foster City, CA, USA) and SYBR Green I Dye (BioRad, Hercules, CA, USA). PCR reactions were performed using the following steps: initial denaturation for 5 min at 95 °C, followed by 40 cycles of 30 s at 95 °C, 45 s at 55–60 °C, 30 s at 72 °C and 72 °C for 5 min as the last step. Triplicates of PCR amplifications were carried out on each plant sample. *Ef1-*α and β-Actin genes were also amplified as reference genes for normalization. The standardized amount of target transcripts was analyzed using relative quantification method (comparative C_t_ method). The C_t_ was used to calculate the eFC in each treated sample with respect to the expression level detected in the corresponding sample under control conditions at the same time point (baseline) using the automated built-in equations of the StepOne™ Software (version 2.1) calculating Δ*R*_*n*_. Calculated eFCs were categorized in two groups upregulated and downregulated genes. As shown in the scale of Fig. 3, each category was divided into five groups going from non-differential genes (In black) to 5 shades of red where the lightest red is monitoring the highest eFC. The same classification of eFC was considered for the downregulation genes but in green color (Fig. 3). The eFC data set was exploited to do GO terms enrichment and co-expression networking.

To test the expression of fungal genes, a standard curve method (diagnostic option) on StepOne™ Software (version 2.1) was performed. The primers used for the quantitative Real-Time RT-PCR are mentioned in Additional file 6: Table S2.

### Gene ontology (GO) annotation using Blast2GO

BLAST analyses were carried out using NCBI-BLASTx, BLASTn and Blast2GO software v3.0.1 [33, 34]. Analyses were performed following the work of Botton’s research group [35]. Blast2GO software utilizes Blast analysis with a user-defined threshold to match on homologous sequences from the NCBI NRPD (nr database). Public available databases are used to search GO association files and retrieve GO annotated terms for the BLAST matches. Databases and files used in the current study were those publicly accessible on October 1st, 2014. The GO annotations of barley sequences were achieved and classified depending on their similarity to genes annotated in plant databases. The GO annotation of barley, rice and arabidopsis genes was derived from TAIR and NCBI [36, 37]. Blast2GO assigns GO term annotations to the query sequence by defining the most explicit annotations based on an annotation rule (AR). The AR works by weighting GO evidence codes for each retrieved GO term (defaults weights: SS = 0.8; ISO = 0.8; ISA = 0.8; ISM = 0.8; IGC = 0.7; IBA = 0.8; IBD = 0.8; IKR = 0.8; IRD = 0.7; RCA = 0.8; IDA = 1.0; IPI = 1.0; IMP = 1.0; IGI = 1.0; IPI = 1.0; IEP = 1.0; EXP = 1.0; TAS = 0.9; NAS = 0.8; C = 0.9; ND = 0.5; IEA = 0.7; NR = 0.0). Considering all precedent parameters, only GO terms were selected only when were greater than a specified AR threshold. BLASTx algorithm was employed with diverse criteria, depending on the length of each input sequence, by regrouped sequences according to their sizes to 3 groups: 0–199 bp, 200–399 bp, ≥ 400 bp. The threshold of BLAST expectation value was repetitively fixed at 10 (e-value = 1.0E-3), while, the HSP length cutoff was fixed at 10, 15, 20 and 33, respectively. This approach permitted high rigors in sequence alignments although for shorter sequences. Annotation of all sequences was carried out by considering specific restrictions for 2 sequence groups according to size in bp, < 200 bp and ≥ 200 bp. GO weight was regularly fixed at 5, the Pre-e-value Hit Filter at 1.0E-3 and the annotation cutoff at 55.

### Enrichment analysis of GO terms, Functional annotation and network analysis

The enrichment analysis was performed in order to annotate the gene function of identified co-expression subnetwork modules. This analysis was carried by TopGO package of Bioconductor using GO annotation data generated from homology search of the *Brachypodium* and *Arabidopsis* proteins. Enriched GO terms in generated modules were summarized in GO Slim terms using Cytoscape software V3.2.1 [38–40], where an InterProScan (IPS) search assisted to GO annotate barley genes using data set of the Badi genome annotation of *Brachypodium* genes [41]. In this analysis, BLASTp was utilized to search and identify homologous sequences in databases taking in consideration a threshold e-value < 1X10E10 between barley query sequences data sets and the protein data sets of each species in the databases. Likewise, a similarity search conducting an NCBI BLAST using BLASTn was performed to identify corresponding transcripts in barley or putative homologous transcript in wheat with a threshold e-value < 1X10E10 against a clustered transcript data set of barley and wheat from the TIGR Gene indices [42]. To permit a more fine matches of shorter sequences (≥200 bp), the similarity threshold was set at 50 %, while for sequences < 200 bp the same threshold was set at 75 %. Afterward, IPS analysis was carried out to find functional GO terms using the appropriate search tool of Blast2GO software [43]. The function of ‘Merge InterProScan GOs to annotations’ was used to enrich annotations number, confirm IPS GOs and distinguish the too general IPS GOs. Finally, the ‘Augment Annotation by ANNEX’ function [44] and the GOslim “goslim_plant.obo”' was applied to distinguish plant-specific GO terms.

In a first step, all genes included in the qPCR analysis were integrated in the analysis for the construction of a baseline co-expression network map using the Blast2GO software v3.0.1 (Additional file 7: Figure S6). The goal of this network was to identify the significant annotation terms and the genes assigned to each GO term. In a second step, subnetwork specific modules were identified from baseline functional network data set using the NeMo plug-in of Cytoscape which helped in predicting possible co-expression network modules [40]. Then, a clustering assessment was elaborated using single linkage method to remove redundancy in all modules with at least one connection. In this case, the network map created using Cytoscape software V3.2.1 [38, 39], is a visual representation of those GO annotation terms and gene assignments. In the constructed network, distinct nodes are used to illustrate each annotation term where is the size of each node is proportional to the number of tested genes assigned to the group. Links between nodes illustrates significant relationship between two annotation terms assigned to the same gene. The link thickness was scaled following the number of shared genes between the two annotation terms (Additional file 7: Figure S6). Cytoscape graph was performed on selected barley ESTs only where the pattern of expression in roots and shoots of resistant genotype (Banteng) was strongly upregulated (*Hv-Pg1, Hv-Pg5, Hv-Pg10, Hv-Pg14, Hv-Pg18, Hv-Pg20, Hv-Pg21, Hv-Pg22, Hv-Pg24, Hv-Pg26, Hv-Pg27, Hv-Pg31, Hv-Pg47, Hv-Pg53* and *Hv-Pg54*). For each subset of selected genes, only the statistically DEGs were included. Here, gene identification was based on its Δ*R*_*n*_ values with either a significant infection-related effect, or a significant domain of interaction. The genes with significant expression change in roots and shoots of each resistance plant were then placed over the baseline function co-expression map. This map format allows a dynamic visualization of the DEGs in terms of the functional effects of their products. Gene subsets that were selected for illustration in Cytoscape maps were those genes enriched in resistant genotype only after 14 or 20 dpi. The process of co-expression and gene networking analysis is illustrated in (Additional file 8: Figure S5).

### Aniline blue staining, trypan blue staining and microscopic analysis

Seedlings were harvested at 14 dpi, chlorophyll removal was performed in 95 % ethanol and stained with trypan blue for hyphae visualization or aniline blue for callose deposition visualization as described in [45], with some modification. Seedlings were incubated for 24 h in 95 % ethanol until all tissues became colorless. After washing in 0.07 M phosphate buffer, samples were incubated for 1–2 h in aniline blue-phosphate buffer (0.01 %) (Sigma, St. Louis) or trypan blue (Sigma, St. Louis). Microscopy analysis was performed on an epifluorescence microscope (Nikon-Japan) with a UV filter (BP 340–380 nm, LP 425 nm). Nikon camera was used to capture bioimages (20 and 40 X) of tissue samples collected 14 dpi with the fungus. Callose deposition intensity was measured on digital photographs using informatics tools of Photoshop software. These tolls allowed calculating the digital pixels in white color relative to the total number of pixels. This calculation was presented par percentage of white/total pixels of the whole surface of each photograph. In some cases when Photoshop software failed to detect a specific callose signal because of significant autofluorescence signal, callose spots were encircled manually for measurement. Average callose deposition intensity was calculated on 10 photographs for each treatment.

### Diagrams, graphical representations and Statistical analyses

Blast2GO output data were utilized in drawing data-pie charts in Microsoft Excel. The principle GO term classes: molecular function, biological process and cellular component were illustrated by a ranking representation of output gene ontology terms. For phylogenetic analysis, all the *HvCsIFs, HvCesAs, HvGTs, HvGSLs, HvCSLAs* genes were aligned using the program ClustalX [46]. Phylogenetic tree was generated and visualized using Treeview 3.2 [47]. For statistical analyses, the qPCR output data were expressed as mean values ± S.E.M and eFC data were calculated. Then, student’s *t* test was applied on eFC data to calculate DEGs meanings. GEGs were considered with a *P* < 0.01 and the range of standard deviation was calculated to consider the error bars in the scale.

### Availability of supporting data

The data sets related to our results are available in the NCBI Sequence expressed sequence tag (EST) repository [54 ESTs: from Hv-Pg1/Accession: JZ845020.1, GI: 847605387/to Hv-Pg54/Accession: JZ845073.1, GI: 847605440/]. All the supporting data of selected DEGS are included as additional files in Additional file 2: Table S1. The remaining data sets of GO terms analysis related to presented results are in figures and additional files of the article (Additional file 9).

## Additional files

Additional file 1: Figure S1. DDRT-PCR approach followed to isolate genes with a resistance-specific expression profile. Seeds of Banteng, Fourat-1 and Thibaut cultivars were inoculated with *P. graminea* - isolate *Sy3*. The extraction of total RNA from plants 14 and 20 dpi was followed by reverse transcription reaction to perform RT-PCRs. The PCR reaction products were profiled on denaturing polyacrylamide gels. PCR products with a resistance and defense-specific profile of expression were selected and calculated. (JPG 82 kb) Additional file 2: Table S1. Homologies of sequences of DD fragments to sequences in the databases (XLSX 15 kb) Additional file 3: Figure S2. Homology of ESTs having R-specific pattern of expression. After sequencing of selected ESTs presented in Table S1, BLASTn and BLASTx algorithms were used for the sequence homology searches against GenBank (nr) and EST (dbEST) databases. This sequence analysis was carried out using at the NCBI websites and then primarily regrouped in five categories of molecular function. (JPG 48 kb) Additional file 4: Figure S3. Homology distribution of selected ESTs across plant species. Sequence homology data were regrouped also in five groups representing the five species listed in homologous data. (JPG 39 kb) Additional file 5: Figure S4. Regrouping of DEGs in different barley genotypes tested in roots and shoots. The Venn diagram shows the number of genes of group A, B and C in roots and shoots tissues in response to *Pg* inoculation at levels of 2 folds or more and a *P* value < 0.05. (JPG 47 kb) Additional file 6: Table S2. Primers used in this work (XLSX 17 kb) Additional file 7: Figure S6. Functional network template of all selected genes (54 ESTs). Selected genes were annotated using Blast2GO and the functional GO terms were manually selected. Network map of GO terms interactions was generated using Cytoscape as a template for the extraction of subnetwork modules. The significance of node size and connections between the nodes is as described in Fig. 7. (JPG 49 kb) Additional file 8: Figure S5. Illustrative and schematics of *in silico* analysis process concerning the co-expression map and gene networking of selected ESTs. (JPG 165 kb) Additional file 9: Supporting data. Go analysis. (XLSX 30 kb)

## Abbreviations

AR: annotation rule
ASE: allele-specific expression
BLAST: basic local alignment search tool
CD: callose deposition
DDRT-PCR: differential display reverse-transcription polymerase chain reaction
DEGs: differentially expressed genes
Dpi: days post-inoculation
eFC: expression fold change
eQTL: expression QTL
ESTs: expressed sequence tags
ETI: effector-triggered immunity
GO: gene ontology
HR: hypersensitive response
Hv: *Hordeum vulgare*
IPS: interproscan
NCBI: National center for biotechnology information
NILs: near isogenic lines
PAL: Phenylalanine ammonia lyase
PAMP: pathogen-associated molecular pattern
Pg: *Pyrenophora graminea*
PRs: pathogenesis-related proteins
PTI: PAMP-triggered immunity
qPCR: quantitative real-time PCR
R: resistant
ROS: reactive oxygen species
RQ: relative quantification
S: susceptible
TDFs: transcript derived fragments
