# Supplementary figures and images for: Deciphering the transcriptional regulation and spatiotemporal distribution of immunity response in barley to Pyrenophora graminea fungal invasion
Source: BMC Genomics. 2016 Mar 22;17:256. doi: 10.1186/s12864-016-2573-x (PMC4804540; doi:10.1186/s12864-016-2573-x)

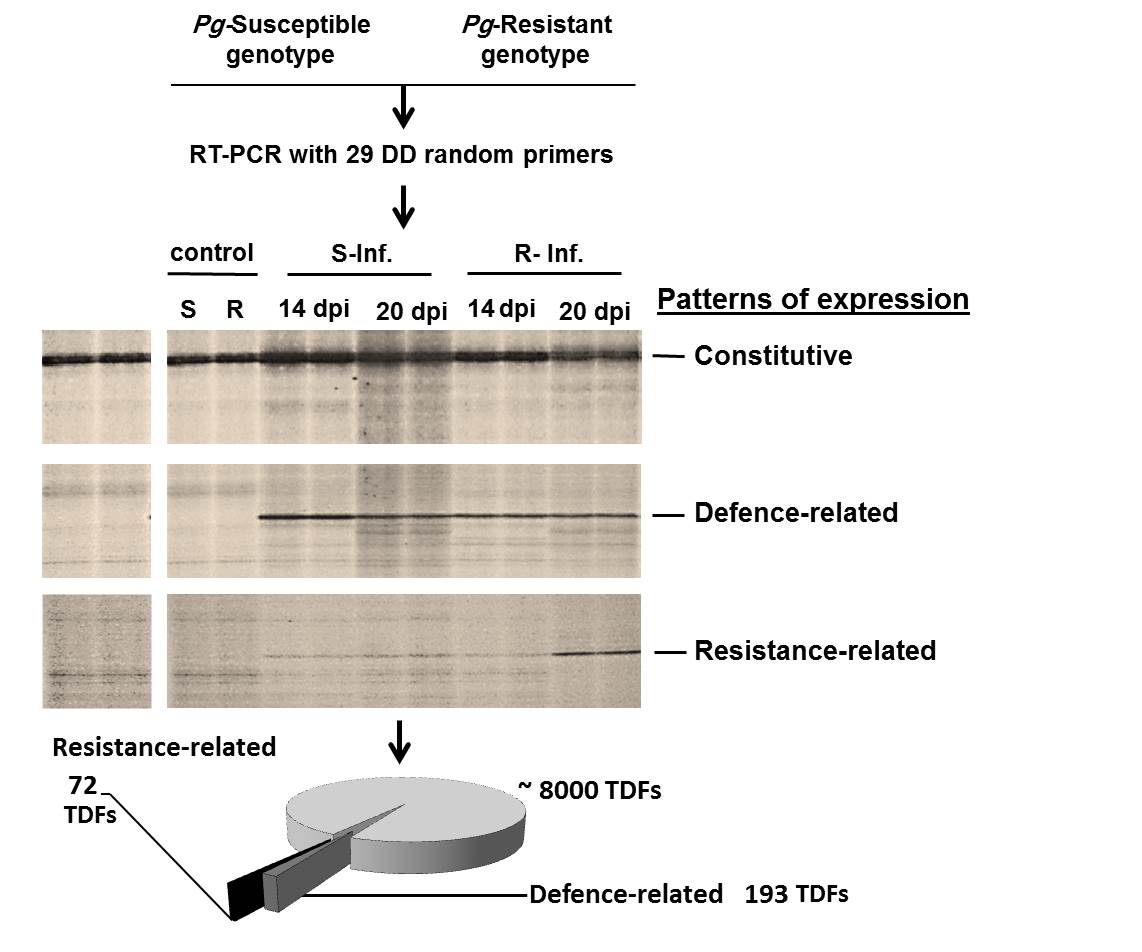

Supplement: Additional file 1: Figure S1. — DDRT-PCR approach followed to isolate genes with a resistance-specific expression profile. Seeds of Banteng, Fourat-1 and Thibaut cultivars were inoculated with P. graminea - isolate Sy3. The extraction of total RNA from plants 14 and 20 dpi was followed by reverse transcription reaction to perform RT-PCRs. The PCR reaction products were profiled on denaturing polyacrylamide gels. PCR products with a resistance and defense-specific profile of expression were selected and calculated. (JPG 82 kb) [file 12864_2016_2573_MOESM1_ESM.jpg]

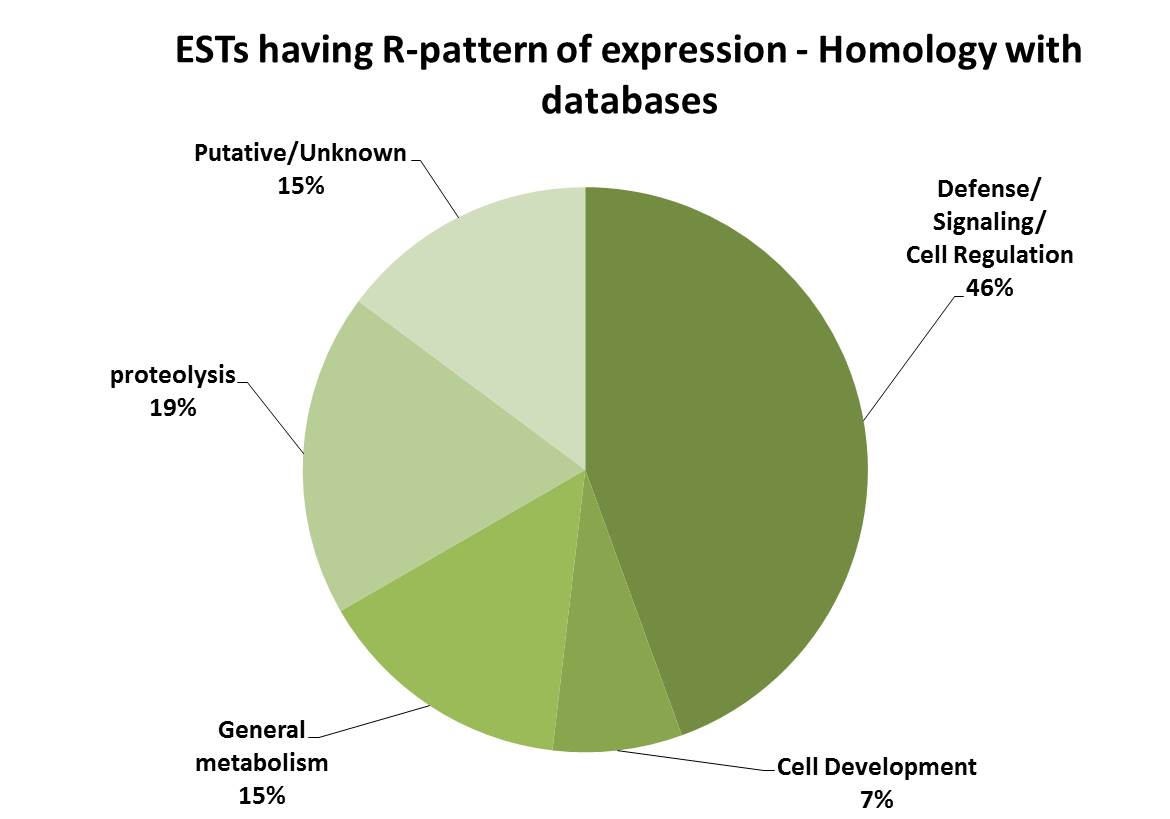

Supplement: Additional file 3: Figure S2. — Homology of ESTs having R-specific pattern of expression. After sequencing of selected ESTs presented in Table S1, BLASTn and BLASTx algorithms were used for the sequence homology searches against GenBank (nr) and EST (dbEST) databases. This sequence analysis was carried out using at the NCBI websites and then primarily regrouped in five categories of molecular function. (JPG 48 kb) [file 12864_2016_2573_MOESM3_ESM.jpg]

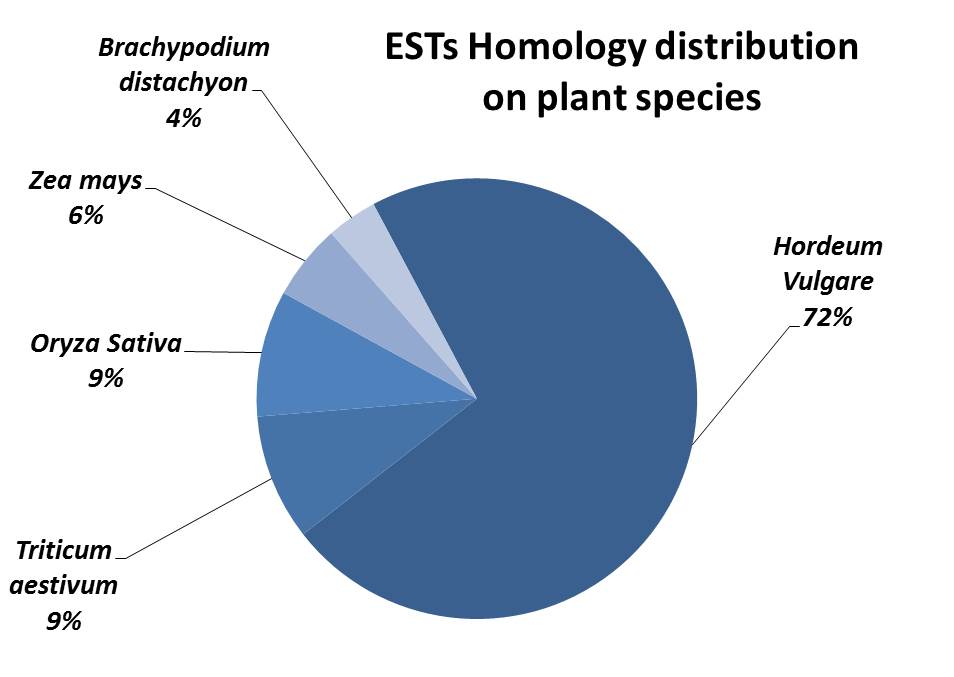

Supplement: Additional file 4: Figure S3. — Homology distribution of selected ESTs across plant species. Sequence homology data were regrouped also in five groups representing the five species listed in homologous data. (JPG 39 kb) [file 12864_2016_2573_MOESM4_ESM.jpg]

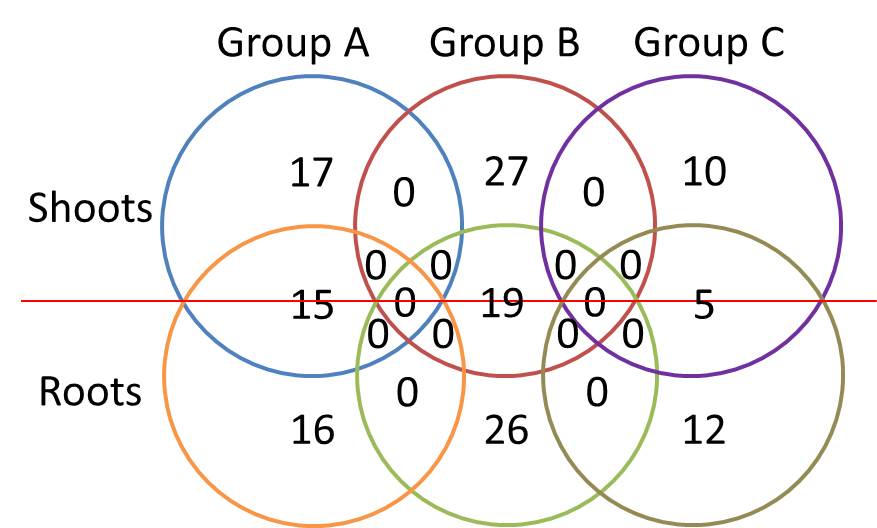

Supplement: Additional file 5: Figure S4. — Regrouping of DEGs in different barley genotypes tested in roots and shoots. The Venn diagram shows the number of genes of group A, B and C in roots and shoots tissues in response to Pg inoculation at levels of 2 folds or more and a P value < 0.05. (JPG 47 kb) [file 12864_2016_2573_MOESM5_ESM.jpg]

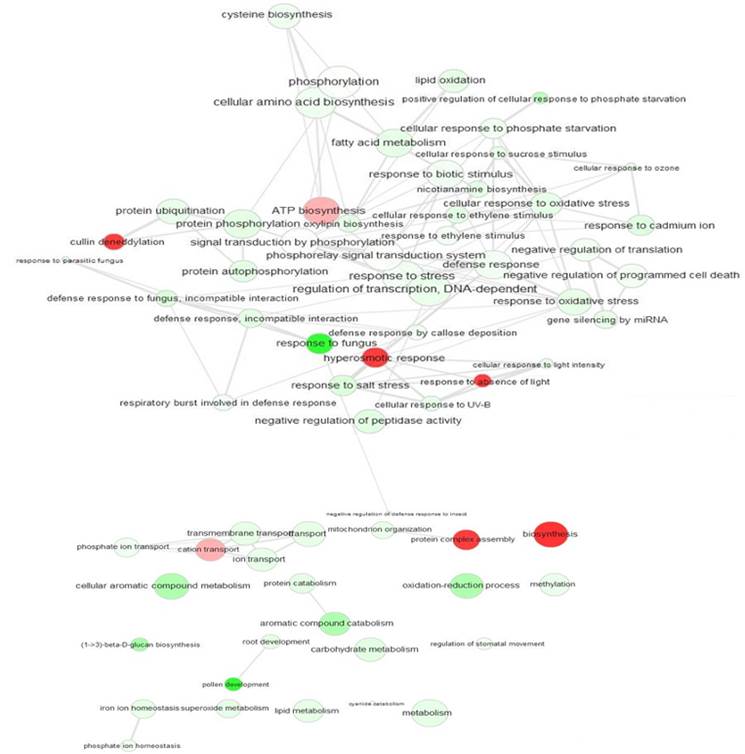

Supplement: Additional file 7: Figure S6. — Functional network template of all selected genes (54 ESTs). Selected genes were annotated using Blast2GO and the functional GO terms were manually selected. Network map of GO terms interactions was generated using Cytoscape as a template for the extraction of subnetwork modules. The significance of node size and connections between the nodes is as described in Fig. 7. (JPG 49 kb) [file 12864_2016_2573_MOESM7_ESM.jpg]

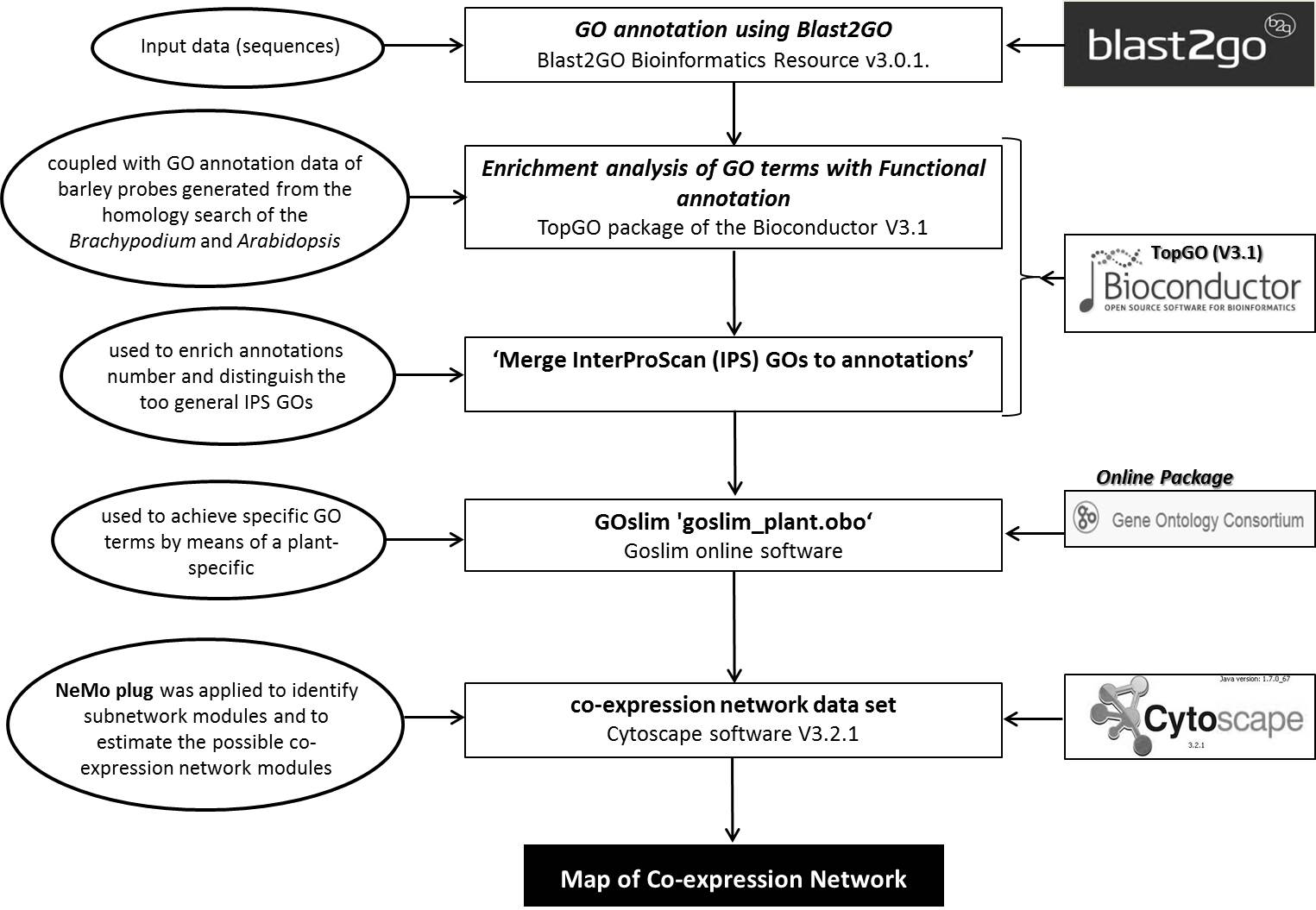

Supplement: Additional file 8: Figure S5. — Illustrative and schematics of in silico analysis process concerning the co-expression map and gene networking of selected ESTs. (JPG 165 kb) [file 12864_2016_2573_MOESM8_ESM.jpg]
